# Changes in neurotransmitter-related functional connectivity along the Alzheimer’s disease continuum

**DOI:** 10.1093/braincomms/fcaf008

**Published:** 2025-01-10

**Authors:** Riccardo Manca, Matteo De Marco, Hilkka Soininen, Livia Ruffini, Annalena Venneri

**Affiliations:** Department of Life Sciences, Brunel University of London, UB8 3PH London, UK; Department of Medicine and Surgery, University of Parma, 43125 Parma, Italy; Department of Life Sciences, Brunel University of London, UB8 3PH London, UK; Institute of Clinical Medicine, Neurology, University of Eastern Finland, 70210 Kuopio, Finland; Nuclear Medicine Division, Azienda Ospedaliero-Universitaria of Parma, 43126 Parma, Italy; Department of Life Sciences, Brunel University of London, UB8 3PH London, UK; Department of Medicine and Surgery, University of Parma, 43125 Parma, Italy

**Keywords:** Alzheimer’s disease, neurotransmitters, functional connectivity, PET, MRI

## Abstract

Alzheimer’s disease may be associated with early dopamine dysfunction. However, its effects on neurofunctional alterations in the neurotransmission pathways remain elusive. In this study, positron emission tomography atlases and functional MRI data for 86 older adults with mild cognitive impairment Alzheimer's disease (MCI), 58 with mild Alzheimer's disease-dementia and 76 cognitively unimpaired were combined to investigate connectivity alterations associated with the dopaminergic and cholinergic systems. A cross-sectional design was used to compare neurotransmitter-related functional connectivity across groups and associations between functional connectivity and cognitive performance. The findings show that the Alzheimer's disease dementia group showed a decline in mesocorticolimbic dopamine-related connectivity in the precuneus but heightened connectivity in the thalamus, whereas the Alzheimer's disease-MCI group showed a decline in nigrostriatal connectivity in the left temporal areas. Acetylcholine-related connectivity decline was observed in both Alzheimer's disease-MCI and Alzheimer's disease-dementia primarily in the temporo-parietal areas. Episodic memory scores correlated positively with acetylcholine- and dopamine-related connectivity in the temporo-parietal cortex and negatively with dopamine-related functional connectivity in the fronto-thalamic areas. This study shows that connectivity alterations in acetylcholine and dopamine functional pathways parallel cognitive decline in Alzheimer's disease and might be a clinically relevant marker in early Alzheimer's disease.

## Teaser

Manca *et al*. report that alterations in functional connectivity associated with the distribution of both dopamine (especially in the mesocorticolimbic pathway) and acetylcholine can be detected in patients with mild cognitive impairment and dementia due to Alzheimer’s disease. Connectivity alterations associated with acetylcholine are also associated with episodic memory decline.

## Graphical Abstract

**Graphical Abstract:**
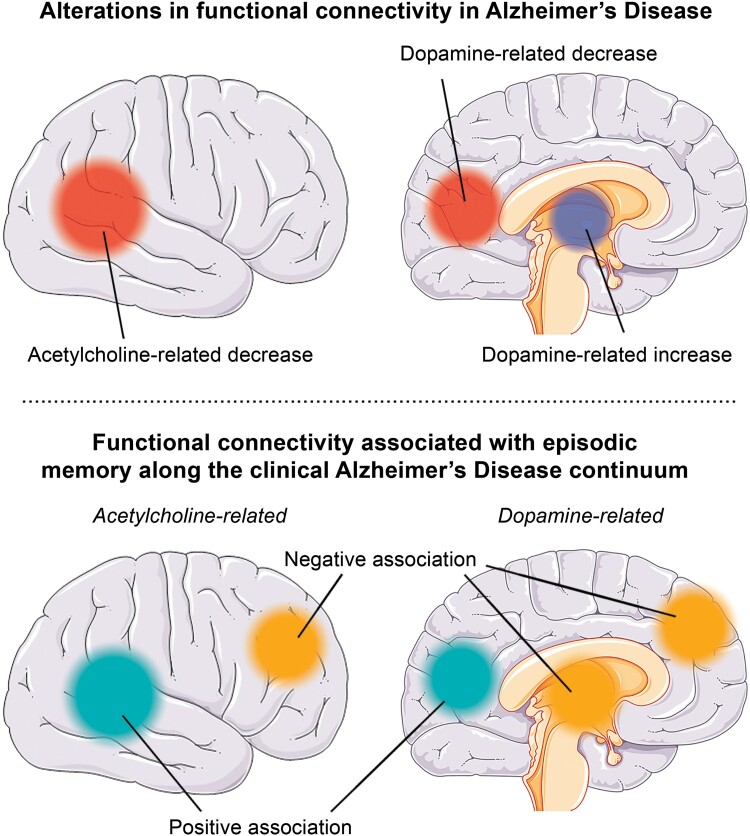

See Zaccone, Nobili, and D'Amelio (https://doi.org/10.1093/braincomms/fcaf057) for a scientific commentary on this article.

## Introduction

Although diagnostic criteria for Alzheimer’s disease (AD) are based on established biological markers, the AT(N) [i.e. amyloid tau (neurodegeneration)] system is flexible, and recognizes the importance of other mechanisms that may contribute to Alzheimer's disease aetiopathogenesis.^1^ Many mechanisms still need to be fully clarified, and more research is needed to detect and characterize them in detail. In this respect, the vulnerability of the ‘isodendritic core’, a set of interconnected brainstem nuclei in the reticular formation, has long been proposed as a major player in a range of neurodegenerative diseases, including Alzheimer's disease.^2,3^ Many of these nuclei send projections to the medial temporal lobe, a territory that is significantly affected by Alzheimer's disease. However, only recently have increasing efforts been made to establish the exact alterations that occur in the brainstem at the pre-symptomatic stages of Alzheimer's disease. A recent neuropathological investigation identified accumulation of cytoskeletal pathology (due to hyperphosphorylated tau protein) across multiple brainstem nuclei at ‘Braak Stage 0’, before any pathological changes are detected in the transentorhinal cortex.^4^ This finding suggests that, although neuronal damage in the basal forebrain and the subsequent dysfunction in acetylcholine (ACh) production have long been established in Alzheimer's disease,^5^ they may not be the primary cause of altered neurotransmission in Alzheimer's disease. Indeed, not all ACh-producing neurons (e.g. those in the brainstem) are heavily affected by Alzheimer's disease pathology.^6^

The role of dopamine (DA) dysfunction in Alzheimer's disease has been investigated in a mouse model expressing a mutated version of the human amyloid precursor protein: neuronal loss in the ventral tegmental area (VTA), but not in the substantia nigra, anticipated alterations in hippocampal neuron function and memory performance.^7,8^ Additional findings have emerged from studies carried out on cognitively unimpaired (CU) older adults and people with Alzheimer's disease. De Marco and Venneri^9^ found that smaller VTA volume values were significantly associated with smaller hippocampal volumes and worse episodic memory performance, particularly in CU older adults. Moreover, lower functional connectivity (FC) between the VTA and left hippocampus was associated with hippocampal atrophy, while episodic memory performance was positively associated with FC between the VTA and mediofrontal areas. Serra *et al*.^10^ found that the FC of the VTA, but not of the locus coeruleus, was progressively less expressed along the Alzheimer's disease continuum and was associated with neuropsychiatric symptoms. In detail, compared with CU, FC between the VTA and the right parietal cortex was weaker in people with mild cognitive impairment (MCI), while a more pronounced FC decline was observed in patients with dementia due to Alzheimer's disease throughout all cortical regions of the posterior default mode network (DMN).

Moreover, a significant alteration in the structural integrity of the VTA, but not of any other brainstem nuclei, was observed in CU older adults before progression to MCI, although no differential rate of longitudinal VTA signal decline was observed between older adults who progressed to MCI and those who remained unimpaired.^11^

The few available MRI studies that have included participants with Alzheimer's disease have important limitations. Volumetric estimates of small brainstem nuclei might be suboptimal, since MRI sequences that are more suitable for visualizing small DA-containing brainstem nuclei, e.g. neuromelanin-sensitive MRI,^12^ have not been consistently used. It is also not clear whether different subcortical/cortical regions that receive projections from the VTA may be equally affected in Alzheimer's disease, and whether the mesocorticolimbic (MCL) pathway may be more vulnerable to early disease processes than the nigrostriatal (NST) pathway, as suggested by a recent positron emission tomography (PET) study.^13^ Moreover, multimodal investigations that combine different neuroimaging modalities are lacking.

To overcome the above-mentioned limitations and to ascertain the potential involvement of DA in the neurofunctional processes underlying Alzheimer's disease, the present study was designed to combine PET atlases that illustrate the distribution of neurotransmitter receptors and transporters with functional MRI to: (1) investigate differences in DA-related FC across the clinical spectrum of Alzheimer's disease (i.e. in three diagnostic groups of CU, MCI due to Alzheimer's disease, i.e. Alzheimer's disease-MCI, and dementia due to Alzheimer's disease, i.e. Alzheimer's disease-dementia); (2) test whether alterations in DA-related FC are associated with worse cognitive performance across groups. ACh-related FC maps were also analysed to investigate the cholinergic system as a second pathway of established relevance in Alzheimer's disease.

## Materials and methods

### Participants

This study was a retrospective analysis of the dataset of the ‘Virtual Physiological Human: DementiA RESearch Enabled by IT’ (VPH-DARE@IT) project. VPH-DARE@IT was a multicentre study funded by the EU (Framework Programme 7, FP7/2007–2013, Grant Agreement no. 601055) with the aim of providing a comprehensive modelling approach to improve the diagnosis and prognosis of dementia. Two hundred twenty participants were included in this study: 86 older adults with a clinical diagnosis of MCI due to Alzheimer’s disease (Alzheimer's disease-MCI),^14^ 58 with dementia due to Alzheimer’s disease (Alzheimer's disease-dementia)^15^ and 76 cognitively unimpaired (CU) controls. The Alzheimer's disease-MCI group included 28 amnestic single-domain, 36 amnestic multi-domain, 16 non-amnestic single-domain and six non-amnestic multi-domain cases. Participants were recruited between December 2013 and June 2017 across three sites: the Institute of Clinical Medicine in Kuopio (Finland), the memory clinic at the Royal Hallamshire Hospital in Sheffield (United Kingdom) and the San Camillo IRCCS Hospital Foundation in Venice (Italy). Patients were approached and invited to take part in this study in a clinical diagnostic setting and mostly recruited at the time of first diagnosis. Eligible patients were provided with informational material about the study if they met inclusion criteria. CU older adults were recruited via leaflets and adverts in the local communities, among patients’ carers and by word of mouth. Participants were selected from the larger sample included in the VPH-DARE@IT project only if the necessary neuroimaging and cognitive data were available. Participants with missing data or with neuroimaging data that did not pass quality control were not considered for inclusion in this study.

All procedures were carried out in compliance with the Declaration of Helsinki and written informed consent was obtained from all participants. Ethical approval was obtained for each sub-cohort from the ethics committee of the Northern Savonia Hospital District (Ref No: 77/2013), the Yorkshire and Humber Regional Ethics Committee (Ref No: 12/YH/0474) and from the ethics committee of the Health Authority Venice and San Camillo IRCCS (Ref No: 2014.08).

The general inclusion and exclusion criteria for the VPH-DARE@IT project have been extensively reported in previous studies.^16^ Briefly, patients with potential non-neurodegenerative causes (e.g. cerebrovascular disease, psychiatric problems and abnormal levels of vitamin B12) of cognitive decline were excluded. Moreover, participants with evidence of medication/substance use known to influence dopaminergic neurotransmission, or with MRI evidence of abnormalities other than the effects of either aging or neurodegeneration, were also excluded. Information regarding ongoing acetylcholinesterase inhibitor (AChEI) treatment and positivity status for both amyloid beta (Aβ) and phosphorylated tau (p-tau), determined in clinical settings by means of any available biomarkers [i.e. cerebrospinal fluid (CSF), PET or blood examination], was also recorded.

Additional inclusion criteria for this study were: (1) availability of all demographic information; (2) availability of good-quality structural (T1-weighted) and functional (T2*-weighted) MRI scans; and (3) availability of at least one cognitive test score common to all three participant cohorts. Although there were no selection criteria about ethnicity and race, this culturally diverse sample included only participants of primarily White Caucasian ethnic background (only one Alzheimer’s disease-dementia patient and one CU control were of an Afro-Caribbean background and one MCI patient and one CU control were of an Indian background).

### Cognitive assessment

For this study, a set of seven cognitive measures common to all cohorts was selected: the logical memory test (immediate and delayed recall),^17^ the letter fluency test (total score, based on language-dependent and clinically established phonological cues),^18^ the similarities test,^17^ the Stroop test (time interference calculated on the 30-item version)^19^ and the digit span test (forwards and backwards scores).^17^

To enable the integration and comparison of the different sub-cohorts, all raw cognitive test scores were transformed into *z*-scores using the means and standard deviations of each cohort’s CU group. A composite score (CS) for global cognitive performance was created by averaging the *z*-scores. The CS was calculated for 215 participants only, as five participants had only three or fewer cognitive measures.

### MRI acquisition

MRI scans were acquired using a shared acquisition protocol across sites on a Philips Achieva 3T scanner in Kuopio and a Philips Ingenia 3T scanner in Sheffield and Venice. All data were collected using the same acquisition parameters that had been harmonized using the ADNI protocol (https://adni.loni.usc.edu/data-samples/adni-data/neuroimaging/mri/mri-scanner-protocols/). The scanning protocol included T1-weighted, T2-weighted, fluid attenuated inversion recovery and T2*-weighted resting-state functional MRI scans.^16^ Structural images were reviewed by a senior neuroradiologist to ensure that the study criteria were met. Only T1-weighted and T2*-weighted images were used in this study. T1-weighted scans were acquired using a magnetization prepared rapid acquisition gradient echo technique (voxel dimensions = 0.94 × 0.94 × 1.00 mm^3^, repetition time = 8.2 s, echo time = 3.8 ms, inversion time = 1 s and flip angle = 8°). T2*-weighted scans were acquired at rest while participants were lying as still as possible with eyes closed (35 axial slices, reconstructed in-plane voxel dimensions = 1.8 × 1.8 mm^2^, slice thickness = 4.0 mm, repetition time = 2.6 s, echo time = 35 ms and number of temporal dynamics = 125).

### MRI pre-processing

Structural and functional MRI data were pre-processed and analysed using SPM12 (Wellcome Centre for Human Neuroimaging, London, UK) running in Matlab R2016b (The Mathworks, Natick, Massachusetts, USA). The CONN toolbox^20^ was used to implement the pipeline, as previously described by our team.^21^ Briefly, the following pre-processing steps were applied: (1) slice-timing and realignment; (2) co-registration of structural and functional images; (3) segmentation of T1-weighted images into grey matter (GM), white matter (WM) and CSF tissue maps; (4) normalization of both T1- and T2*-weighted scans into the MNI space; and (5) smoothing of both images via a Gaussian kernel of 6 mm. Multiple denoising steps were carried out on the pre-processed T2*-weighted images: (1) regressing out the first five components of WM and CSF signals (by means of aCompCor); (2) regressing out 12 motion parameters (three rotations, three translations and their first derivative); (3) application of a band-pass filter (0.008–0.1 Hz) to remove non-neural signals; (4) linear de-trending; and (5) de-spiking.

Subsequently, native-space GM, WM and CSF maps were used to extract tissue volumes (in ml) using the ‘get_totals’ script. The volumes of the three tissues were summed to calculate total intracranial volume (TIV) for each participant.

To obtain ACh- and DA-related FC maps, all pre-processed functional scans were fed into an additional pre-processing pipeline called receptor-enriched analysis of functional connectivity by targets (REACT).^22^ First, five publicly available PET atlases were sourced: D1 DA receptor,^23^ D2 DA receptor,^24^ vesicular ACh transporter (VAChT),^25^ M1 muscarinic receptor^26^ and α_4_β_2_ nicotinic receptor.^27^ A single-photon emission computerized tomography (SPECT) atlas was used for the DA transporter (DAT).^28^ All of the atlases were resampled to resize the voxel dimensions of the pre-processed functional scans (i.e. 2 × 2 × 2 mm^3^), this was a deviation from the original REACT pipeline. Subsequently, the following atlas-specific reference regions were masked out: the cerebellum for three atlases,^23,24,26^ the occipital lobe for the atlas by Dukart *et al*.^28^ and WM for the atlas by Aghourian *et al*.,^25^ whereas Hillmer *et al*.^27^ used no reference region methods. Finally, all atlases were normalized by setting the minimum value to zero and by rescaling the new values of each image within the range defined by the minimum and maximum values.

Before extracting the neurotransmitter-related FC maps, a set of masks was created: six atlas-specific masks obtained by intersecting all functional scans with each PET atlas and a publicly available GM mask (https://github.com/ottaviadipasquale/react-fmri), and a seventh, atlas-independent mask resulting from the sole intersection of all functional scans with the GM mask. These masks were used in two sequential steps at the basis of the FC extraction procedure.^22^ First, PET/SPECT atlases were used as spatial regressors to estimate participant-specific time series in the blood-oxygen-level-dependent signal fluctuations weighted by the distribution of the target neurotransmitter receptor/transporter across the whole brain. An independent model was used for each atlas. Second, the estimated time series were used as temporal regressors to extract whole-brain neurotransmitter-related FC spatial maps for each participant. The same functional scans (resolution: 2 × 2 × 2 mm^3^) were used in both steps, deviating from the original REACT pipeline.

Furthermore, the above-mentioned modified REACT pipeline was re-run to study the DA-related FC of the MCL and NST pathways separately by using specific GM masks. To do this, 2 binary GM masks were created using the WFU PickAtlas toolbox, and combining the following, literature-informed regions:^29-31^ (1) nucleus accumbens, amygdala, hippocampus, entorhinal cortex (i.e. BA28/34), parahippocampal gyrus, cingulate gyrus, subcallosal gyrus (i.e. BA25), lateral orbitofrontal cortex, lateral and medial superior frontal gyrus, middle frontal gyrus and orbital inferior frontal gyrus were combined as MCL afferents; (2) caudate nucleus, putamen and globus pallidus were combined as NST afferents. These masks were used to extract FC maps between each DA system and the entire brain.

### Statistical analysis

Demographic variables, cognitive scores and global MRI volumes were compared across groups using ANOVA and Kruskal–Wallis test for normally and non-normally distributed variables, respectively. The Shapiro–Wilk test was used for preliminary checks of the normality of distributions. The χ^2^ test was used for categorical variables. A significance threshold of *P* < 0.05 was used for all analyses.

ANOVA models were implemented in SPM12 to compare neurotransmitter-related connectivity maps across all groups, and independent-sample *t*-tests were used for pairwise *post hoc* comparisons. Age, education, sex, TIV and recruitment site were included as covariates in all models, and a significance cluster-forming threshold of *P* < 0.001 with family wise error correction to account for multiple comparisons was used.

Finally, using SPM12, multiple regression models were used to test the association between all neurotransmitter-related FC maps and both the CS of global cognitive performance (except for five participants with three or fewer cognitive scores) and the logical memory test delayed-recall score (LM-DR, except for seven participants who did not complete this test). The same covariates and significance threshold used in the between-group comparisons were applied to these regression models.

A sensitivity analysis was carried out with SPM12 to test the above-mentioned models in participants with biomarker data and comparisons were performed between Aβ− CU (*n* = 28), Aβ+ Alzheimer's disease-MCI (*n* = 13), and Aβ+ Alzheimer's disease-dementia (*n* = 16) groups, while regression models were tested on all Aβ+ participants (*n* = 30, namely 13 Alzheimer's disease-MCI, 16 Alzheimer's disease-dementia and 1 CU). Due to the limited sample size of groups with biomarkers, a significance cluster-forming threshold of *P* < 0.005 was used.

## Results

The patient and control groups were matched for age, but CU controls had significantly more years of education than the Alzheimer's disease-MCI and Alzheimer's disease-dementia groups (Table 1). Both patient groups showed worse performance than CU in all cognitive tests, with the only exception of scores on the Digit Span forwards that did not differ between Alzheimer's disease-MCI and CU. Moreover, the proportions of diagnostic groups differed across centres (Table 1), with considerably fewer Alzheimer's disease-dementia patients recruited in Venice. Some participants in both patient groups were on treatment with AChEI at the time of recruitment, with a significantly higher proportion in the Alzheimer's disease-dementia group (46%) than in the Alzheimer's disease-MCI group (7%).

**Table 1:** Clinical profiles of the participant groups (values are means and standard deviations and statistical models are ANOVAs unless otherwise specified)

Variable	CU (*n* = 76)	AD-MCI (*n* = 86)	AD-dementia (*n* = 58)	*F*	*P*
Age^a^	69.5 (12.3)	72.5 (15.8)	68.0 (17.5)	2.07^b^	0.355
Education	13.6 (3.3)	**11.4 (3.7)**	**11.4 (3.4)**	9.82	<0.001
Sex (M/F)^c^	29/47	36/50	28/30	1.39^d^	0.499
MMSE^a^	29.0 (1.2)	**27.0 (3.0)**	**21.0 (6.7)**	97.26^b^	<0.001
LM-IR	0.0 (1.0)	**−1.8 (1.4)**	**−3.2 (1.3)**	129.23	<0.001
LM-DR	0.0 (1.0)	**−2.4 (2.0)**	**−4.8 (2.0)**	147.82	<0.001
PF	0.0 (1.0)	**−0.6 (1.3)**	**−1.2 (1.1)**	21.28	<0.001
ST—time	0.0 (1.0)	**1.5 (2.4)**	**1.6 (3.6)**	17.45	<0.001
DS-F	0.0 (1.0)	−0.4 (1.0)	**−0.7** (**1.1)**	8.27	<0.001
DS-B	0.0 (1.0)	**−0.7 (0.8)**	**−1.1 (1.1)**	17.94	<0.001
Similarities	0.0 (1.0)	**−1.2 (1.5)**	**−2.4 (2.0)**	36.51	<0.001
CS	0.0 (0.5)	**−0.8** (**0.8)**	**−1.7 (0.9)**	82.39	<0.001
TIV	1452.1 (166.2)	1441.3 (139.2)	1451.9 (152.0)	0.14	0.871
GMV^a^	612.3 (99.9)	**600.3** (**91.9)**	**548.5** (**114.0)**	21.25^b^	<0.001
AChEI (n. treated)	0	**6**	**27**	63.04^d^	<0.001
Site (S1/S2/S3)^c,e^	18/37/21	43/27/16	27/28/3	20.6^d^	<0.001

In bold, significant differences in the *post hoc* tests comparison with unimpaired controls.

AChEI, acetylcholinesterase inhibitors; AD, Alzheimer’s disease; CS, composite score; CU, cognitively unimpaired; DR, delayed recall; DS-F/B, digit span—forwards/backwards; F, females; GMV, grey matter volume; IR, immediate recall; LM, logical memory; M, males; MCI, mild cognitive impairment; MMSE, mini mental state examination; PF, phonemic fluency; S1/S2/S3, site 1/2/3; ST, Stroop test; TIV, total intracranial volume.

^a^Values are medians and interquartile ranges.

^b^Kruskal–Wallis test.

^c^Values are frequencies.

^d^χ^2^ test.

^e^Site 1 = Kuopio; Site 2 = Sheffield; Site 3 = Venice.

The Aβ positivity status was available for 81 participants (Kuopio and Sheffield sites): 60 CSF, 2 PET and 19 blood examinations. For the 60 participants with CSF data, p-tau positivity status was also available (Table 2). No biomarker data were available for the Venice site.

**Table 2:** Biomarker status profiles of the participant groups (frequencies)

Variable	CU	AD-MCI	AD-dementia	*χ* ^2^	*P*
Aβ status	*n* = 29	*n* = 20	*n* = 32	23.04	<0.001
Aβ+	1	**13**	**16**		
Aβ−	28	**7**	**16**		
p-tau status	*n* = 18	*n* = 19	*n* = 23	8.94	0.011
p-tau+	2	**10**	**12**		
p-tau−	16	**9**	**11**		

In bold, significant differences in the *post hoc* tests comparison with unimpaired controls.

Aβ, amyloid beta; AD, Alzheimer’s disease; CU, cognitively unimpaired; p-tau, phosphorylated tau.

Both groups of patients were more likely to be positive for both Aβ and p-tau biomarkers than the CU group.

**Figure 1:**
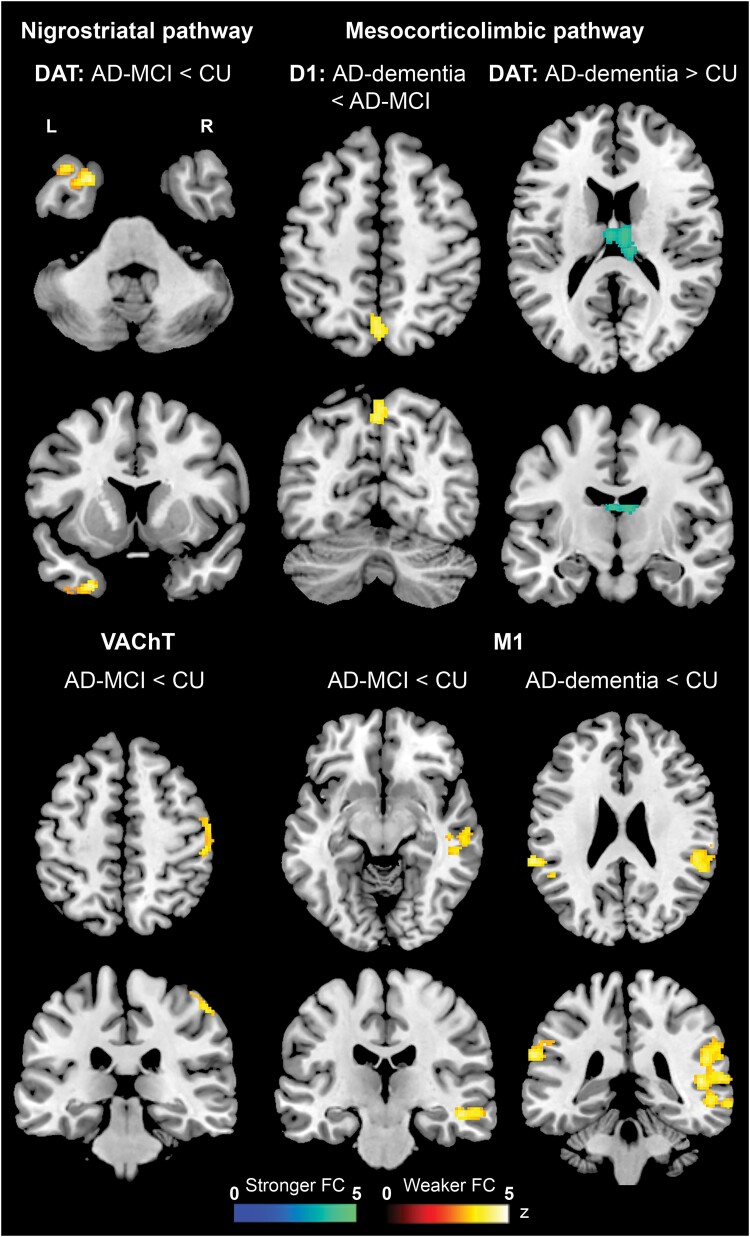
**Voxel-based group differences in FC.** Differences between CU, Alzheimer's disease-MCI and Alzheimer's disease-dementia groups (*t*-tests) in neurotransmitter-enriched FC across different receptor pathways (significance cluster-forming threshold of *P* < 0.001 with family-wise error correction); L, left; R, right.

Two ANOVA models revealed significant group differences: DAT-related FC in the right insula and thalami, and M1-related FC in the right superior/middle temporal gyri and in the left inferior parietal lobule (IPL) (Table 3). Additional pairwise models revealed DA-related FC alterations only from the analyses of the MCL and NST pathways. In particular, the Alzheimer's disease-dementia group had lower MCL D1-related FC than the Alzheimer's disease-MCI group in the left precuneus but higher MCL DAT-related FC than CU in the bilateral thalamus (dorsolateral and mediolateral nuclei). Instead, the Alzheimer's disease-MCI group had lower NST DAT-related FC in the left superior temporal gyrus than the CU group. Compared with CU, lower M1-related FC was observed in Alzheimer's disease-MCI in a cluster limited to the right superior/middle temporal gyri, and this also extended to both IPLs in Alzheimer's disease-dementia (Fig. 1 and Table 3). Finally, VAChT-related FC was also found to be lower in the right postcentral (BA 2 and 3) and precentral (BA 6) gyri in the Alzheimer's disease-MCI group than in the CU group.

**Table 3:** Differences in neurotransmitter-enriched FC across groups

Contrast		Cluster size	Side	Brain region	*t* value	MNI coordinates		
	*P*-value					*x*	*y*	*z*
**Mesocorticolimbic pathway: Dopamine—D1**								
AD-MCI < AD-dementia	0.017	144	L	Precuneus (BA7)	4.71	−2	−63	49
			L	Precuneus (BA7)	4.47	0	−69	54
**Mesocorticolimbic pathway: Dopamine—DAT**								
ANOVA^a^	0.010	72	R	Insula (BA13)	14.08	30	16	13
	0.003	92	R	Thalamus	12.72	6	−18	18
			L	Thalamus	9.93	−2	−14	19
AD-dementia > CU	0.004	156	R	Thalamus	4.36	6	−18	18
			L	Thalamus	4.28	−2	−14	19
**Nigrostriatal pathway: Dopamine—DAT**								
AD-MCI < CU	0.031	134	L	STG (BA38)	4.67	−25	9	−39
			L	STG (BA38)	4.20	−39	15	−38
**Acetylcholine—M1**								
ANOVA^a^	<0.001	688	R	STG (BA22)	14.59	61	−43	10
			R	MTG (BA21)	13.50	55	−35	−5
			R	MTG (BA21)	12.98	57	−25	−9
	0.007	125	L	IPL (BA40)	12.30	−61	−42	33
			L	IPL (BA40)	11.46	−65	−38	27
AD-MCI < CU	0.001	371	R	STG (BA2)	4.42	47	−22	−13
			R	STG (BA22)	4.33	63	−45	6
			R	MTG (BA21)	4.18	59	−18	−11
AD-dementia < CU	<0.001	1008	R	STG (BA22)	5.40	59	−43	10
			R	MTG (BA21)	4.73	59	−25	−9
			R	STG (BA22)	4.68	49	−39	7
	0.007	261	L	IPL (BA40)	4.78	−65	−38	27
			L	IPL (BA40)	4.25	−53	−46	31
**Acetylcholine—VAChT**								
AD-MCI < CU	0.017	236	R	Precentral gyrus (BA6)	4.15	43	−9	62
			R	Postcentral gyrus (BA2)	3.92	49	−29	55
			R	Postcentral gyrus (BA3)	3.79	53	−15	55

AD, Alzheimer’s disease; BA, Brodmann area; CU, cognitively unimpaired; D1, D1 dopamine receptor; DAT, dopamine transporter; IPL, inferior parietal lobule; L, Left; M1, M1 muscarinic acetylcholine receptor; MCI, mild cognitive impairment; MTG, middle temporal gyrus; R, right; STG, superior temporal gyrus; VAChT, vesicular acetylcholine transporter.

^a^ *F* values are displayed for ANOVA.

**Figure 2:**
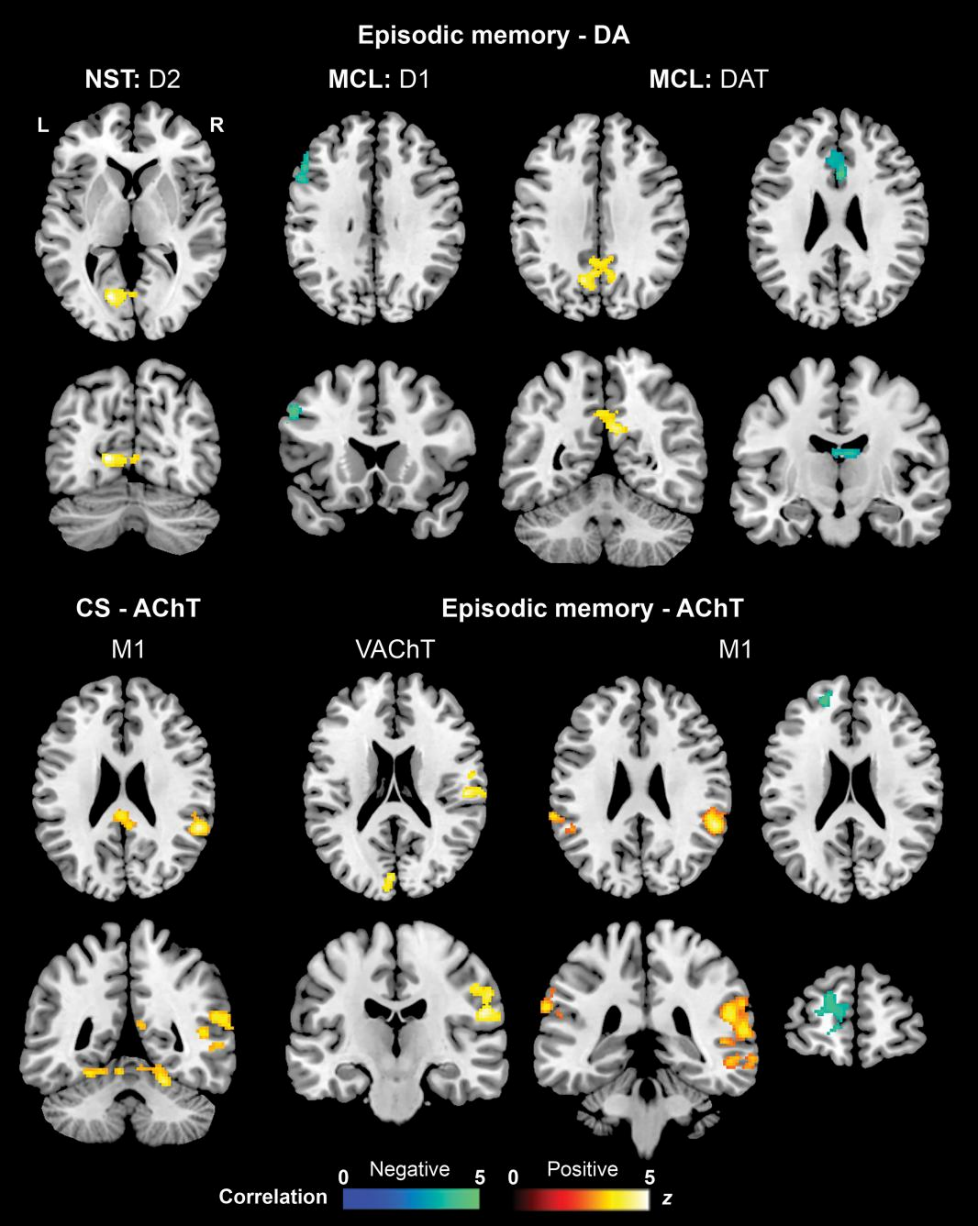
**Voxel-based FC-cognition associations across groups.** Associations (multiple regressions) between neurotransmitter-enriched FC and episodic memory performance in dopaminergic and cholinergic receptor pathways across the whole sample (including CU, Alzheimer's disease-MCI and Alzheimer's disease-dementia; significance cluster-forming threshold of *P* < 0.001 with family wise error correction). CS, composite score; L, left; MCL, mesocorticolimbic; NST, nigrostriatal; R, right.

Regression analysis found that the cognitive CS was positively associated with M1-related FC in the right superior/middle temporal, right cerebellar (culmen) and bilateral posterior cingulate areas (Fig. 2 and Table 4). Variegated associations were observed between episodic memory performance and FC. Indeed, in the MCL pathway, LM-DR scores were negatively associated with D1-related FC in left middle/inferior frontal cortices and DAT-related FC in bilateral anterior cingulate and thalamus (i.e. the dorsolateral nucleus), and positively associated with DAT-related FC in the bilateral precuneus/posterior cingulate. Moreover, episodic memory performance was also negatively associated with NST D2-related FC in the left occipito-temporal area. For the cholinergic system, LM-DR scores were positively associated with VAChT-related FC in the right postcentral gyrus and left occipito-temporal areas and with M1-related FC in the bilateral inferior parietal and right superior/middle temporal cortices. However, a negative association was also found with M1-related FC in left-lateralized superior/middle frontal areas (Table 4).

**Table 4:** Associations between neurotransmitter-enriched FC and both global cognitive and episodic memory performance

Association		Cluster size	Side	Brain region	*t* value	MNI coordinates		
	*P*-value					*x*	*y*	*z*
**Cog-CS**								
**Acetylcholine—M1**								
Positive	<0.001	1148	R	STG (BA13)	5.12	55	−44	22
			R	STG (BA41)	4.73	47	−39	11
			R	MTG (BA21)	4.48	53	−35	−5
	0.026	213	R	PCC (BA29)	4.95	7	−45	15
			R	PCC (BA31)	4.07	5	−40	27
			L	PCC (BA23)	4.02	−9	−32	29
	0.001	389	R	Cerebellum—culmen	4.53	21	−50	−20
			R	Cerebellum—culmen	4.41	17	−54	−14
			R	Cerebellum—culmen	4.03	3	−56	−10
**Episodic memory**								
**Mesocorticolimbic pathway: Dopamine—D1**								
Negative	0.015	168	L	MFG (BA9)	4.74	−52	19	36
			L	MFG (BA46)	4.09	−50	28	27
			L	IFG (BA45)	3.55	−58	13	22
**Mesocorticolimbic pathway: Dopamine—DAT**								
Positive	<0.001	405	R	PCC (BA31)	4.58	5	−51	28
			L	Precuneus (BA7)	4.51	−11	−63	35
			L	PCC (BA31)	4.43	−21	−62	21
Negative	0.001	354	R	ACC (BA24)	5.77	2	19	29
			L	ACC (BA32)	4.33	−4	36	23
			R	ACC (BA32)	4.01	14	18	39
	0.012	213	R	Thalamus	4.77	4	−18	18
			L	Thalamus	4.20	−2	−12	19
**Nigrostriatal pathway: Dopamine—D2**								
Positive	0.028	173	L	Cuneus (BA23)	5.06	−15	−73	6
			L	Lingual gyrus (BA18)	4.00	−7	−71	4
			L	RSC (BA30)	3.75	1	−69	4
**Acetylcholine—M1**								
Positive	<0.001	1159	R	IPL (BA40)	5.93	57	−44	22
			R	STG (BA22)	4.77	61	−43	10
			R	MTG (BA22)	4.31	59	−47	2
	0.035	222	L	IPL (BA40)	4.51	−65	−36	29
			L	IPL (BA40)	4.16	−53	−46	31
			L	IPL (BA40)	3.72	−59	−38	39
Negative	0.026	241	L	SFG (BA10)	4.16	−21	50	24
			L	MFG (BA10)	3.96	−11	59	13
			L	SFG (BA10)	3.87	−17	58	21
**Acetylcholine—VAChT**								
Positive	0.017	285	L	Cuneus (BA23)	4.75	−5	−77	11
			L	Cuneus (BA18)	4.18	−5	−82	19
			L	Lingual gyrus (BA18)	3.38	−9	−67	2
	0.016	291	R	Postcentral gyrus (BA43)	4.54	53	−20	21
			R	Postcentral gyrus (BA43)	4.32	57	−6	16
			R	Postcentral gyrus (BA2)	4.10	57	−22	33

ACC, anterior cingulate cortex; BA, Brodmann area; D1, D1 dopamine receptor; D2, D2 dopamine receptor; DAT, dopamine transporter; IFG, inferior frontal gyrus; IPL, inferior parietal lobule; L, left; M1, M1 muscarinic acetylcholine receptor; MFG, middle frontal gyrus; MTG, middle temporal gyrus; PCC, posterior cingulate cortex; R, right; RSC, retrosplenial cortex; SFG, superior frontal gyrus; STG, superior temporal gyrus; VAChT, vesicular acetylcholine transporter.

**Figure 3:**
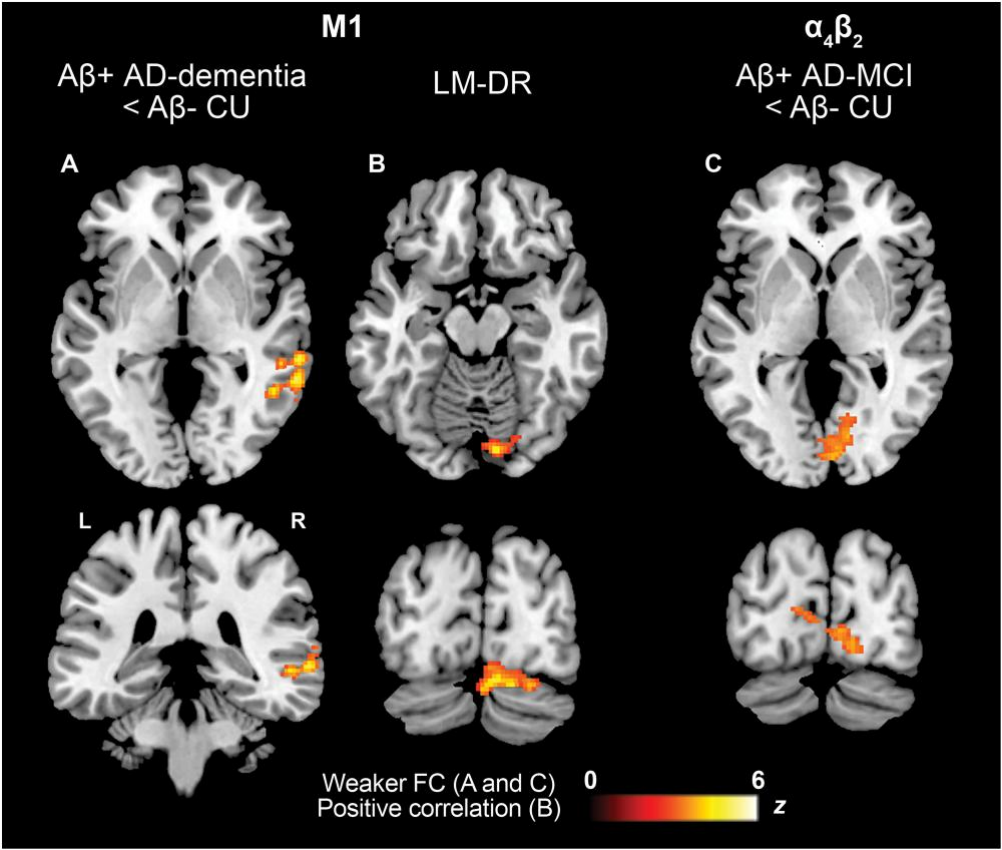
**Sensitivity analysis.** This figure shows differences (*t*-tests; **A** and **C**) in neurotransmitter-enriched FC between Aβ+ patients (Alzheimer's disease-MCI and Alzheimer's disease-dementia) and Aβ− CU older adults and associations (multiple regressions; (**B**) between neurotransmitter-enriched FC and episodic memory (LM-DR) in all Aβ+ participants (including CU, Alzheimer's disease-MCI and Alzheimer's disease-dementia; significance cluster-forming threshold of *P* < 0.005 with family wise error correction); L, left; R, right.

Some of the above findings were replicated in the sensitivity analyses. M1-related FC in the right posterior middle temporal gyrus (MTG) was lower in the Aβ+ Alzheimer's disease-dementia group than in the Aβ− CU group (Fig. 3 and Table 5). Episodic LM-DR scores were positively correlated with M1-related FC in a cluster comprising the right fusiform gyrus and the right cerebellum in Aβ+ participants. A novel finding emerged that was not highlighted in the main analysis: lower α_4_β_2_-related FC in Aβ+ Alzheimer's disease-MCI than in the Aβ− CU group in the right lingual gyrus and the left posterior cingulate cortex (PCC) (Fig. 3 and Table 5). No between-group differences or cognition-FC associations were found in the DA systems.

**Table 5:** Results of the sensitivity analysis

	Cluster size	Side	Brain region	*t* value	MNI coordinates		
*P*-value					*x*	*y*	*z*
**Acetylcholine—α_4_β_2_: Aβ+ AD-MCI < Aβ− CU**							
0.013	354	R	Lingual gyrus (BA18)	3.57	11	−77	3
		R	Lingual gyrus (BA17)	3.57	5	−89	−3
		L	Posterior cingulate cortex (BA 30)	3.38	−1	−71	8
**Acetylcholine—M1: Aβ+ AD-dementia < Aβ− CU**							
0.027	330	R	Middle temporal gyrus (BA 21)	4.29	63	−45	−4
		R	Middle temporal gyrus (BA 22)	4.24	63	−35	−1
		R	Middle temporal gyrus (BA 21)	3.88	53	−35	−3
**Acetylcholine—M1: positive association with LM-DR in Aβ+ participants**							
0.010	349	R	Cerebellum-declive	5.36	7	−80	−19
		R	Fusiform gyrus (BA 19)	4.86	37	−70	−21
		R	Cerebellum-declive	4.60	16	−82	−21

AD, Alzheimer’s disease; BA, Brodmann area; CU, cognitively unimpaired; L, left; M1, M1 muscarinic acetylcholine receptor; LM-DR, logical memory—delayed recall; MCI, mild cognitive impairment; R, right.

## Discussion

This study found that, along with ACh-related FC alterations, DA-related FC changes may be detected in both the Alzheimer's disease-MCI and Alzheimer's disease-dementia groups only when dopaminergic pathways were investigated separately. However, when analyses were restricted to patients with available evidence of Alzheimer's disease pathological changes (i.e. Aβ+ status), the only neurofunctional alterations evident in both patient groups were those associated with the cholinergic system.

In the MCL pathway, the Alzheimer's disease-dementia group showed lower D1-related FC than the Alzheimer's disease-MCI group in the left precuneus and higher DAT-related FC than the CU group in the thalamus. The former finding suggests a decline in FC between the target areas of the MCL pathway and the posterior DMN in Alzheimer's disease-dementia mediated by the distribution of the D1 receptor. This seems to be in line with a previous observation of weaker FC between the VTA and the posterior DMN.^10^ In fact, the DMN includes a posterior hub comprising the precuneus and the PCC^32^ that has long been established to be dysregulated in Alzheimer's disease.^33,34^ It is possible that the FC decline observed in the clinical progression from Alzheimer's disease-MCI to Alzheimer's disease-dementia might be mediated by dopaminergic dysfunction evident between the MCL system and the posterior DMN. Although no data are available for Alzheimer's disease, the cerebral distribution of the D1 receptor has been linked to the gradient-based brain organization, particularly to greater functional differentiation between the DMN and the sensory cortices.^35^ The findings of the present study suggest a breakdown in the communication between the MCL system and the DMN. In line with this view, the regression analyses showed a positive association between MCL DAT-related FC in the posterior DMN and episodic memory performance, the most severely affected function in the Alzheimer's disease clinical continuum. However, the cross-sectional design of this study prevented any conclusions from being drawn on the dynamic evolution of FC in this pathway over time. Moreover, D1-related FC alterations emerged only when the Alzheimer's disease-dementia group was compared with Alzheimer's disease-MCI participants, but not when compared with the CU group. Therefore, it cannot be ruled out that these findings might be a consequence of divergent sub-threshold FC differences between the two patient groups and CU older adults (i.e. higher FC at the Alzheimer's disease-MCI stage and lower FC at the Alzheimer's disease-dementia stage).

DAT-related FC, by contrast, was heightened between the MCL system and the thalamus in the Alzheimer's disease-dementia group when compared with CU controls. This was found in the laterodorsal nucleus and, to a lesser extent, the mediodorsal nucleus of the thalamus bilaterally. The laterodorsal nucleus is connected primarily with the restrosplenial, mediotemporal and occipital areas,^36^ is involved in memory functions and is particularly atrophic in Alzheimer's disease.^37^ In contrast, the mediodorsal nucleus is connected to the temporal and prefrontal areas and is typically affected by Alzheimer's disease pathology to a lesser extent than the laterodorsal nucleus.^38^ As episodic memory performance was negatively associated with DAT-related FC between the MCL system and both these thalamic nuclei and the anterior cingulate cortex, such alterations are suggestive of maladaptive plasticity.^39^ Similar negative associations between the LM-DR scores and D1-related FC in left middle and inferior frontal areas further support this interpretation. Alternatively, it may be possible that heightened MCL DA-related FC in fronto-thalamic areas, known to be involved in executive processes,^40^ plays a temporary compensatory role by supporting memory pathways in an attempt to cope with inefficient retrieval in people with MCI and mild Alzheimer's disease-dementia. Indeed, recent evidence has accumulated to suggest a prominent role of the mediodorsal nucleus in regulating frontal lobe activation when required over an extended period of time.^41^

These findings seem to contradict those of De Marco and Venneri,^9^ who found that stronger FC between the VTA and prefrontal areas was associated with better episodic memory. However, there are some methodological differences between the two studies that may limit comparisons of findings. In contrast to De Marco and Venneri,^9^ this study investigated FC weighted using specific PET atlases and focused on the cortical and subcortical targets of the MCL pathway rather than on the VTA. It cannot be ruled out, however, that degeneration of the VTA may induce divergent FC changes in the VTA itself and in the cerebral regions innervated by its projections.

The findings related to the NST system were, overall, inconsistent. Lower DAT-related FC was observed in the left temporal pole in the Alzheimer's disease-MCI group than in the CU group but was not replicated in the Alzheimer's disease-dementia group. In contrast, the regression models indicated a positive association between episodic memory scores and D2-related FC in the retrosplenial and temporo-occipital regions.^42,43^ Although heterogeneous, these results suggest that clinical Alzheimer's disease stages may also result in alterations in NST DA-related FC in left-lateralized areas known to support verbal memory.^44,45^

The above-mentioned findings were not replicated in sensitivity analyses carried out on the patient groups with evidence of Aβ+ status. There are two main reasons for this lack of replication. First, DA pathways may be altered in Alzheimer's disease but to a lesser extent than in the cholinergic system.^2^ DA dysfunction could either be a secondary process or occur at a later stage in the aetiopathogenesis of Alzheimer's disease compared with ACh alterations. Second, the small number of participants with available biomarkers strongly limited the sample size of the groups included in the sensitivity analysis that may have been underpowered to detect small effects.

In contrast, neurofunctional alterations in the cholinergic system appeared to be more robust, especially those related to the M1 receptor. When compared with CU controls, the Alzheimer's disease-MCI group had lower M1-related FC in the right-lateralized temporal areas, while the Alzheimer's disease-dementia group showed lower FC in the bilateral temporo-parietal cortices, including the IPL that is part of the DMN.^32^ These findings were replicated in the sensitivity analysis, although lower M1-related FC in the right MTG was only observed in the Aβ+ Alzheimer's disease-dementia group when compared with the Aβ− CU group. This pattern suggests that gradual worsening of M1-related FC in posterior temporal areas may parallel the clinical progression of Alzheimer's disease, since better global cognition and episodic memory performance were positively associated with M1-related FC in the temporal, inferior parietal and posteromedial areas, bilaterally. In Aβ+ participants, this association persisted in a smaller cluster between the right fusiform gyrus and the decline of the cerebellum. Previous studies have found reduced M1 receptor density in patients with Alzheimer's disease, compared with CU older adults, primarily in medio-posterior temporal and limbic areas and associated with reduced perfusion and longer disease duration.^46-49^ All these sources of evidence suggest that cholinergic dysfunction mediated by the M1 receptor is evident in areas heavily affected by Alzheimer's disease pathology such as the posterior and inferior temporal regions.

VAChT-related FC was lower among individuals with Alzheimer's disease-MCI than among CU controls in a small right-lateralized sensorimotor cluster. Episodic memory was also positively associated with higher VAChT-related FC in the right somatosensory cortex, but also with regions identified by the analysis of dopaminergic pathways, i.e. the left occipito-temporal cortex and left BA23. The clinical relevance of this finding, however, is uncertain, since sensorimotor areas are neither rich in VAChT^25^ nor particularly affected by Alzheimer's disease pathology and the findings have not been replicated in the sensitivity analysis. Given the relative sparing of sensory neural structures at the disease stages investigated in the present study, these findings might reflect compensatory over-recruitment of these neural structures in support of neighbouring failing systems to support behavioural performance.

Lower α_4_β_2_-related FC in Aβ+ Alzheimer's disease-MCI than in the Aβ− CU group in the right lingual gyrus and the left PCC was only found in the sensitivity analysis, thus further strengthening the suggestion that ACh depletion can trigger malfunctioning in the posterior temporo-parietal cortices clinically relevant to Alzheimer's disease. As for VAChT-related FC, this result also remains difficult to interpret because no differences were found between the Alzheimer's disease-dementia and CU groups. This could be due to a lack of statistical power given the smaller sample size of the Alzheimer's disease-dementia group compared with the Alzheimer's disease-MCI group. A possible alternative explanation could be that a considerable number of people with Alzheimer's disease-dementia were treated with AChEIs (27 out of 58). Such pharmacological treatment might have induced adjustments in the cholinergic system to such an extent that it reduced alterations in resting-state activity secondary to ACh depletion, as found in previous studies of patients treated with Donepezil.^50,51^ However, the small number of treated patients and the lack of longitudinal MRI data for this sample prevent any more specific investigation of the nature of these effects, unbiased by unknown variables.

The convergence of findings in this study suggests that clinical severity of Alzheimer's disease is primarily associated with FC decline in the posterior DMN and in temporal areas (typically affected by Alzheimer's disease pathology) and, potentially, with FC increase in fronto-thalamic areas. Research on CU adults found that the basal forebrain (i.e. the main source of ACh) suppresses DMN activation, while the mediodorsal thalamic nucleus increases it.^52^ As the lack of DMN deactivation has been extensively documented in Alzheimer's disease,^53^ FC alterations associated with the distribution of the M1 receptor may represent clinically informative resting-state fMRI markers of Alzheimer's disease severity. M1 receptor density is maximal in the ventral striatum, innervated by the VTA,^30,31^ and in the temporal and posterior DMN areas.^26,54^ Additionally, the M1 receptor is functionally relevant to memory processes^55^ and its dysregulation appears to be linked to Alzheimer's disease pathological processes.^56^ Alterations in the M1/M4 receptor distribution may be associated with the duration of cognitive symptoms, while symptom severity in Alzheimer's disease appears to be accounted for by neurofunctional changes in the temporal and posterior DMN areas.^46^ The M1 receptor appears to be an ideal candidate target for potential treatments aimed at slowing the progression of cognitive decline in Alzheimer's disease whose mechanism of action could be easily tested by means of the method utilized in this study (i.e. REACT). Similarly, MCL DAT-related FC may also offer potential insights into Alzheimer's disease pathological changes since MCL DAT reductions have been reported to be more severe than in the NST pathway^13^ and lower CSF levels of DAT have been associated with hypometabolism in the posterior DMN in Alzheimer's disease.^57^

This study had some limitations. First, PET scans were not available, thus preventing any conclusion on the potential impact of Alzheimer's disease-induced neurotransmitter alterations on brain function. Second, not all receptors for the DA and ACh neurotransmitters could be assessed because of the current lack of established PET ligands. Furthermore, overlaps between the neural territories innervated by ACh- and DA-producing nuclei are well-known, but the risk of confounding effects is minimized by the use of receptor- and transport-specific voxel-based density maps. Third, this study focused only on DA and Ach; however, other neurotransmitters are affected by Alzheimer's disease pathological processes, such as noradrenaline. In fact, the locus coeruleus seems to be vulnerable to hyperphosphorylated tau accumulation in preclinical stages of Alzheimer's disease.^58^ Fourth, the design of this study was cross-sectional, thus limiting the interpretation of these findings regarding the longitudinal evolution of neurofunctional alterations across the Alzheimer's disease continuum. Fifth, only a proportion of the patients included in this study had Alzheimer's disease biomarkers to support clinical diagnoses, as patients were recruited before the publication of the AT(N) research framework.^1^ Particular care was taken to ensure that, not only did they meet criteria for clinical diagnosis of Alzheimer's disease-MCI^14^ or Alzheimer's disease-dementia,^15^ but also that they all had an extensive follow-up indicating clinical progression in line with an Alzheimer's disease aetiology. Finally, although culturally diverse, the sample of this study was predominantly ethnically homogenous. Future studies should aim to improve inclusivity practices to enable the recruitment of diverse and representative cohorts to improve replication and generalizability of these findings.

This study represents the first investigation of neurotransmitter-related functional alterations in individuals with MCI and mild dementia due to Alzheimer's disease. These findings suggest that combining *a priori* knowledge of the distribution of specific neurotransmitters (via PET atlases) with advanced MRI processing techniques may lead to the generation of clinically informative biomarkers for Alzheimer's disease. Such biomarkers may be useful to track symptom changes (both in cognition and behaviour) over time, characterize specific phenotypes (e.g. one study applied REACT to characterize the neural correlates of cognitive fatigue in people with multiple sclerosis^59^) and provide insights for treatment targets. Indeed, previous studies have highlighted how the REACT technique can detect pharmacologically induced FC changes related to specific neurotransmitter systems known to be modulated by specific medications (e.g. 3,4-methylenedioxymethamphetamine and serotonin,^22^ methylphenidate and DA/noradrenaline transporters,^60^ and lysergic acid diethylamide and DA and serotonin^61^). Such FC changes were also associated with drug-evoked behavioural responses.

Furthermore, this approach might have prognostic implications in those CU individuals who are biomarker-positive but do not manifest any subjective or objective symptoms of cognitive change. An additional application of this approach would aid in distinguishing individuals with MCI who are likely to progress to dementia due to Alzheimer's disease from those with MCI caused by other conditions. Future research should also address whether confounding factors, such as lifestyle (e.g. type of diet), drug use and smoking, might have a significant impact on neurotransmitter-related FC in order to disentangle the effects of neurodegeneration from those of modifiable risk factors. A further fruitful development would be the design of much-needed hypothesis-driven neuroimaging outcome measures to test the mechanisms of action of new pharmacological interventions for Alzheimer's disease. This appears to be particularly timely in the new era of disease-modifying treatments as well as to explore new avenues towards a cure for Alzheimer's disease.

## Data Availability

The data supporting the findings of this study are available from the corresponding author, upon reasonable request.
